# Employment and working conditions and risk of suicidal behaviors: a systematic review and meta-analysis of longitudinal studies

**DOI:** 10.5271/sjweh.4308

**Published:** 2026-09-01

**Authors:** Linda L Magnusson Hanson, Ida EH Madsen, Sandra Blomqvist, Rebecka Holmgren, Kathrine Sørensen, Reiner Rugulies

**Affiliations:** 1 Division of Psychobiology and Epidemiology, Department of Psychology, Stockholm University, Sweden.; 2 National Research Centre for the Working Environment, Copenhagen, Denmark.; 3 National Institute of Public Health, University of Southern Denmark, Denmark.; 4 Section of Epidemiology, Department of Public Health, University of Copenhagen, Denmark.; 5 Department of Public Health Sciences, Stockholm University, Sweden.

**Keywords:** organizational, psychosocial work environment, suicide, suicide attempt

## Abstract

**Objectives:**

Systematic research on employment/working conditions as risk or protective factors for suicidal behaviors is limited. In this study, we systematically reviewed the literature on a range of employment or working conditions and the risk of suicidal behaviors (suicide and suicide attempt), focusing on longitudinal studies.

**Methods:**

We searched Web of Science, Scopus, PsycInfo, and Embase during 1995–2024 for cohort and case-control studies and performed meta-analysis to pool data if there were ≥2 studies on the same type of conditions and outcome.

**Results:**

A total of 27 studies met the eligibility criteria, of which 14 studies were used in meta-analyses. Meta-analyses showed statistically significant associations between part-time work [relative risk (RR) 1.21, 95% confidence interval (CI) 1.12–1.30], job loss (RR 2.27, 95% CI 1.12–4.61), job insecurity (RR 1.64, 95% CI 1.36–1.96), high job demands (RR 1.14, 95% CI 1.08–1.20), low job control (RR 1.11, 95% CI 1.05–1.17), job strain (RR 1.32, 95% CI 1.29–1.36), workplace bullying (RR 1.78, 95% CI 1.16–2.73), and suicide. In terms of the of evidence, we found moderate and low certainty for job loss and job insecurity, respectively. For other working conditions, certainty of evidence was considered very low or could not be assessed because of too few studies. Studies investigating suicide attempts were limited, and we only found very low evidence for workplace bullying and suicide attempt (RR 1.33, 95% CI 1.09–1.62, in a previously published meta-analysis). For all other conditions, we could not assess certainty of evidence of associations with suicide attempt.

**Conclusions:**

Although some working conditions may be risk factors for suicide, the number of studies is still too limited to allow for certainty of evidence on associations between specific working conditions and suicide.

Suicidal behaviors, referring to behaviors that may result in the ending of one's life, either fatal (suicide) or non-fatal but with an intent to die (suicide attempts), continue to represent major public health concerns (1). It is estimated that more than 700 000 individuals globally take their lives annually (2), and roughly 20 times as many make an attempt to take their life (3). Consequently, a considerable proportion of all deaths among young-to-middle-aged individuals are attributed to suicide (4), with substantial emotional and economic consequences for individuals, families, organizations and society.

Although mental health disorders are key risk factors for suicidal behaviors, social determinants may play an important role, either independently or in interaction with prevalent mental disorders and underlying genetic, biological, psychological, and psychosocial determinants (1). There is some evidence for unemployment as a social determinant of suicidal behavior (5–7), and that psychosocial working conditions, such as low job control, may be associated with the risk of suicide (8). However, only a limited number of systematic reviews on working conditions (referring to any employment, organizational or work characteristics influencing how individuals experience their work environment) and suicidal behaviors have been published. Existing reviews have also had a restricted focus on psychosocial working conditions/job stressors or specific social stressors and have thus covered a limited set of working conditions, such as job demands, control and support (9), workplace bullying and violence (10–12). Furthermore, the systematic reviews published by Leach et al (10) and Milner et al (8) primarily relied on cross-sectional studies. To date, systematic research on working conditions and suicidal behavior thus remains limited, hampering suicide prevention initiatives focusing on modifiable factors in the workplace (13, 14). Consequently, there is a need for insights into a broader range of working conditions based on a growing body of literature since 2018. More knowledge about specific working conditions constituting risk or protective factors for suicidal behaviors could open new prevention strategies.

With this article, we respond to the call for systematically examining the role of working conditions in the etiology of suicidal behaviors (13, 14). We conducted a systematic review and meta-analysis aiming to review and synthesize the current epidemiological literature on the associations between working conditions and risk of suicidal behavior, focusing on longitudinal studies on a wide range of potential work-related risk and protective factors.

## Method

### Information sources and search strategy

This review was performed in line with the Preferred Reporting Items for Systematic reviews and Meta-Analyses (PRISMA) guidelines and followed a preregistered protocol in PROSPERO (2022 CRD42022301704). During 11–17 January 2022, we searched the databases Web of Science (including MEDLINE), Scopus, PsycInfo, and Embase for original studies in English which had been published between 1 January 1995 and 31 December 2021. To incorporate the most recent evidence, we updated the results through a top-up search on 5 December 2024. This search, based on the same databases and search terms, covered literature published between 1 January 2022 and 5 December 2024. A combination of search terms for work/organization, suicidal behaviors, and study design was used to identify (i) observational studies with a prospective or retrospective design such as cohort, case–control studies and mortality studies and (ii) intervention studies. More details about the search strategy and search terms are provided in the supplementary material (www.sjweh.fi/article/4308) Appendix 1. Previous reviews were also screened for additional relevant publications.

### Eligibility criteria

We used the following Population, Exposure, Comparator, Outcomes, and Study design (PECOS) criteria to identify eligible studies. (i) Population comprised individuals of working age 15–65 years; (ii) Exposure was reported for associations between at least one working condition and the outcome(s) of interest. As delineated in the introduction, we defined working conditions as any employment, organizational- or work characteristic that influences how individuals experience their work environment. We excluded, however, physical working conditions as measures were too heterogeneous for the purpose of this review. We also excluded studies using unemployment status or occupational title as exposure variable, unless occupation was used as the basis for an assessment of working conditions, for example by applying a job exposure matrix; (iii) The study used a relevant comparison group (e.g. compared a group of exposed to a group the unexposed, cases to controls, or exposed to the general population); (iv) Outcome included any type of incident suicidal behavior, defined as “behaviors that may result in ending one’s life, whether fatal or not” (including both suicide and suicide attempt but excluding suicidal ideation, as suicidal ideation is not necessarily associated with intent to die or actions and similar risk factors) (1); (v) We included observational studies, with a prospective, retrospective or a case–control design, and intervention studies. We excluded studies with a cross-sectional design.

### Selection and data collection

Two independent authors from the research team screened titles and abstracts of retrieved records using Rayyan. Any discrepancies between reviewers were solved by consensus. Next, full-text screening was performed to determine if the studies fulfilled all the inclusion criteria, and the main reason for exclusion was recorded. Studies that did not present estimates for the associations of interest, including confidence intervals (CI) or P-values from univariate or multivariate analyses, were also excluded in this stage. Finally, physical working conditions were excluded due to large heterogeneity in exposures, leaving employment and psychosocial working conditions in focus for the review. The principal author extracted information which a second author checked. Separate estimates for suicide and suicide attempts were extracted whenever available, since predictors of the two may differ (15). In case a study presented several risk estimates, we selected the estimate that was adjusted for several sociodemographic characteristics such as sex and age, and indicators of socioeconomic status.

### Risk of bias assessment

Two independent authors assessed risk of bias in the included studies according to the Newcastle-Ottawa Scale (NOS) for non-randomized studies, and any differences in opinion were resolved though consensus (For more details on the extraction and NOS scale see the supplementary appendix). Based on NOS, we regarded the overall quality of a study as: (i) good if the study obtained 3–4 stars in the selection domain, 1–2 stars in the comparability domain and 2–3 stars in the outcome/exposure domain; (ii) fair if the study obtained 2 stars in the selection domain, 1–2 stars in the comparability domain and 2–3 stars in the outcome/exposure domain, and (iii) poor if the study obtained 0–1 stars in the selection domain or 0 stars in the comparability domain, or 0–1 stars in the outcome/exposure domain (16).

### Synthesis

Following prior reviews on mental health-related outcomes encompassing a wide range of working conditions (17, 18), the exposures were grouped into job demands, job control, job strain (a dichotomized measure of high demands and low control), effort–reward imbalance, workplace support, workplace justice, workplace conflicts, workplace bullying (considered as unwanted/unacceptable behaviors being threatening, hostile, humiliating, or degrading which are repeated and long-lasting), workplace threats/violence (considered as any unwanted/unacceptable behaviors being threatening, hostile, humiliating, or degrading (19, 20), shift work, reduced working time (ie, part-time work, defined as working hours <35 hours/week), long working hours (>40 hours/week), job insecurity [usually considered as a subjective threat of job loss or a concern for continued employment (21)], or additionally into job loss, precarious work [considered as irregular, poorly paid, insecure, unprotected employment (22) such as agency work, temporary work, casual/on-call work, self-employment], sexual/gender based harassment (considered as unwanted/unacceptable behaviors being threatening, hostile, humiliating, or degrading of a sexual nature), or other working conditions.

Meta-analyses were performed if there were ≥2 studies on the same type of exposure and outcome, except for workplace bullying, since the studies we identified had been synthesized in a meta-analysis elsewhere (12). The meta-analyses combined presented risk estimates, including odds ratio (OR), hazard ratio (HR), relative risk (RR) or incidence rate ratio (IRR), into a pooled estimate of RR. Estimates based on continuous measures of employment or working conditions were pooled separately from those using a dichotomous classification (applicable to job demands only). The meta-analyses were fixed-effects meta-analyses performed with R package metafor, based on the inverse variance method (23, 24). This approach was used because the number of studies in meta-analyses were small (<5 studies), and because there is substantial uncertainty in estimates of I^2^ and funnel plot asymmetry with small numbers of studies, we refrained from reporting such estimates of heterogeneity or indications of reporting bias (24, 25).

### Assessment of certainty of evidence

Through consensus, two authors assessed the certainty in the body of evidence using GRADE (Grading of Recommendations Assessment, Development, and Evaluation) (26). The GRADE approach is a structured way of rating the certainty of evidence as high, moderate, low, or very low. The initial study quality is considered low in GRADE for observational studies, but it can be upgraded (due to large effects, findings of dose–response gradient or all plausible confounding would reduce the demonstrated effect or increase the effect if no effect was observed) or downgraded (due to risk of bias, inconsistency of results, indirectness of evidence, imprecision or publication bias) (27).

## Results

### Study selection

**Figure 1:**
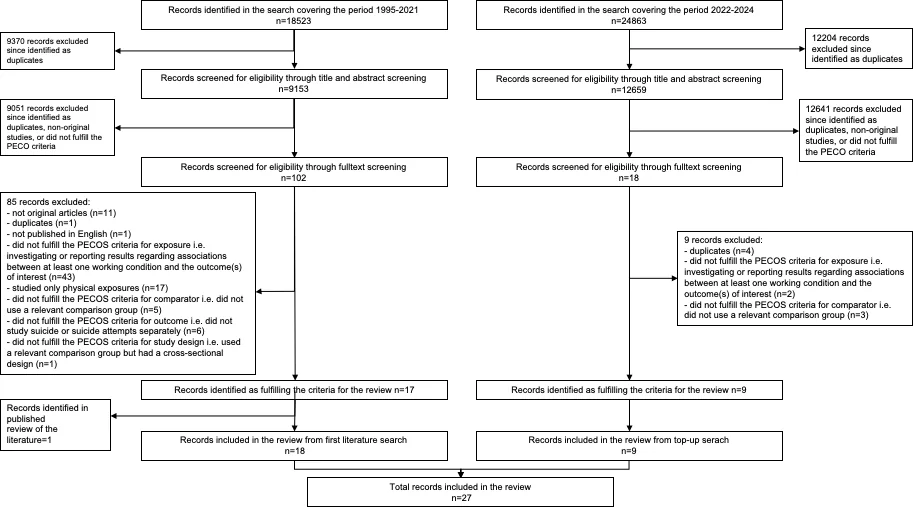
Flowchart describing identification of records, screening and inclusion of studies in the systematic review.

The first literature search until 31 December 2021 resulted in 18 523 records and 9153 after exclusion of obvious duplicates, of which 17 original articles were identified for the review according to the PECOS criteria (figure 1). The top-up search from 1 January 2022 to 5 December 2024 resulted in an additional 12 659 records for screening, of which 9 articles were included in the review. We identified 1 additional study in a previous review, yielding a total of 27 eligible studies published between 1995 and 2024 (see figure 1 and supplementary table S1).

### Study characteristics

Of the 27 included studies, 18 (67%) were cohort studies, while the remaining 9 (33%) were case–control studies. Most studies were from high income countries from Europe (Austria, Belgium, Denmark, Finland, France, Germany, Italy, Norway, Sweden, and the UK), North America (Canada, US), Asia (China, Japan, Korea) and Oceania (Australia and New Zealand). The studies largely varied regarding populations and sample sizes, with some studies being based on entire working populations (with maximum 4 458 673 cohort members) and others on specific populations according to region, organization, or occupation (the smallest study including 84 cases and 35 controls). Most of the reviewed studies assessed suicide separately (N=24), whereas some assessed suicide attempts separately (N=10), and one study assessed suicide attempts together with suicide. In total, 14 studies (providing 33 risk estimates) were considered eligible for meta-analysis.

### Risk of bias

Of the included studies, 18 (67%) were considered of good quality (with low risk of bias), 3 (11%) of fair quality, and 6 (22%) of poor quality (with high risk of bias) (see tables 1 and 2 for a summary of overall risk of bias and supplementary table S1 for details).

**Table 1:** Summary of studies in the review addressing associations between employment or working conditions and suicide, including their main characteristics, presented risk estimates adjusted for covariates, and overall study quality (according to Newcastle-Ottawa Scale). [CI=confidence interval; HR=hazard ratio.]

Exposure and study	Country	Study design	Study sample	Sample size	Risk estimates	Quality
**Shift work**						
Baumert et al 2014 (33)	Germany	Cohort	General population	6817	HR (95% CI) for chronobiological exposures (overtime, shift work, night shifts, taskwork, assembly-line work) 2.32 (1.01–5.35)	Good
Niedhammer et al 2022 (53)	France	Cohort	General working population	1 511 456	HR (95% CI) for shift work daytime; men and women respectively: 1.39 (1.19–1.63) and 1.06 (0.78–1.44) HR (95% CI) for night work; men and women respectively: 0.97 (0.81–1.16) and 0.95 (0.42–2.14) HR (95% CI) for shift work and night work; men and women respectively: 1.38 (1.19–1.61) and 1.98 (0.28–14.14)	Good
**Part-time work**						
Guseva-Canu et al 2021 (34)	Switzerland	Cohort	General working population (men)	1 534 564	RR (95% CI) for part-time work (1–35h/week) 1.26 (1.12–1.42)	Good
Lee et al 2020 (54)	Korea	Cohort	General working population	14 484	HR (95% CI) for part-time work (<35h/week) 0.95 (0.11–8.39)	Fair
Wild et al 2021 (55)	Switzerland	Cohort	General working population (women)	1 771 940	RR (95% CI) for part-time work (1–35 h/week) 1.18 (1.07–1.29)	Good
**Long working hours**						
Guseva-Canu et al 2021 (34)	Switzerland	Cohort	General working population (men)	1 534 564	RR (95% CI) for working hours >45h/week 0.95 (0.87–1.03), and unknown working hours 1.56 (1.25–1.94)	Good
Lee et al 2020 (54)	Korea	Cohort	General working population	14 484	HR (95% CI) for working hours 45–52h/week 3.89 (1.06–14.29), and >52 h/week 3.74 (1.03–13.64)	Fair
Wild et al 2021 (55)	Switzerland	Cohort	General working population (women)	1 771 940	RR (95% CI) for working hours >45 h/week 1.13 (0.95–1.33)	Good
**Job loss**						
Hoyer et al 2009 (56)	Denmark	Case–control	General population	170	IRR (95% CI) for job loss 2.94 (1.16–7.46)	Good
Kolves et al 2006 (57)	Estonia, Germany	Case–control	General population	638	P<0.001, but OR for job loss could not be calculated	Poor
Milner et al 2014 (58)	Australia	Case–control	General population	307	OR (95% CI) for job loss 1.60 (0.54–4.75)	Good
**Precarious work**						
Schneider et al 2011 (36)	Germany	Case–control	General population	559	OR (95% CI) for temporary employment 2.3 (0.8–6.8), and self-employment 0.9 (0.4–2.1)	Poor
**Job insecurity**						
Almasi et al 2009 (59)	Hungary	Case–control	General population	120	OR f (95% CI) or concern over work prospects 5.90 (3.02–11.53)	Good
Blomqvist et al 2022 (60)	Sweden	Cohort	General working population	65 571	HR (95% CI) for job insecurity; all, men and women respectively: 1.49 (1.01–2.20), 1.29 (0.77–2.17) and 1.90 (0.97–3.73)	Good
Law et al 2014 (61)	China	Case–control	General working population	177	OR for “chronic impact from job insecurity” ^a^1.46 (P=0.028)	Poor
**Job demands**						
Almroth et al 2022 (62)	Sweden	Cohort	General working population	3 017 962	HR (95% CI) for high demands; men and women respectively: 0.80 (0.71–0.90) and 1.00 (0.83–1.21)	Good
Milner et al 2017 (63)	Australia	Case–control	General population	23 017	OR (95% CI) for high job demands; men and women respectively: 1.36 (1.26–1.46) and 0.81 (0.72–0.92)	Good
Niedhammer et al 2021 (29)	France	Cohort	General working population	1 496 332	HR (95% CI) for high job demands; men and women respectively: 1.05 (0.91–1.21) and 1.27 (0.98–1.65).	Good
Ostry et al 2007 (30)	Canada	Case–control	Specific sector/occupation (men)	648	OR (95% CI) for higher levels of demands; 0.78 (0.69–0.89)	Good
Pan et al 2023 (28)	Sweden	Cohort	General working population	4 458 673	HR (95% CI) for job demands; men and women respectively: medium–low trajectory: 0.81 (0.76–0.87) and 1.08 (0.93–1.26), medium–high trajectory 0.72 (0.66–0.78) and 0.87 (0.73–1.05), high trajectory 0.76 (0.67–0.86) and 1.03 (0.81–1.33)	Good
Schneider et al 2011 (36)	Germany	Case–control	General population	559	OR (95% CI) for higher levels of time pressure 0.9 (0.7–1.2), and for higher levels of responsibility 1.4 (1.1–1.8)	Poor
Tsutsumi et al 2007 (64)	Japan	Cohort	General population (men)	3125	RR for high job demands 0.73 (0.22–2.38)	Fair
**Job control**						
Almroth et al 2022 (62)	Sweden	Cohort	General working population	3 017 962	HR (95% CI) for low job control, men and women respectively: 1.43 (1.25–1.63) and 1.30 (1.08–1.58),	Good
Milner et al 2017 (63)	Australia	Case–control	General population	23 017	OR (95% CI) for low job control; men and women respectively: 1.35 (1.26–1.44) and 0.91 (0.80–1.02)	Good
Niedhammer et al 2021 (29)	France	Cohort	General working population	1 496 332	HR (95% CI) for low job control men and women respectively: 1.18 (0.99–1.42) and 1.14 (0.78–1.65)	Good
Pan et al 2023 (28)	Sweden	Cohort	General working population	4 458 673	HR (95% CI) for job control; men and women respectively: medium–high trajectory 1.35 (1.24–1.47) and 1.20 (1.04–1.39), medium–low trajectory 1.49 (1.36–1.63) and 1.54 (1.32–1.80), low trajectory 1.49 (1.33–1.68) and 1.32 (1.10–1.60)	Good
Schneider et al 2011 (36)	Germany	Case–control	General population	559	OR (95% CI) for higher levels of monotonous work 1.5 (1.1–2.0)	Poor
Tsutsumi et al 2007 (64)	Japan	Cohort	General population (men)	3125	RR for low job control 4.10 (1.31–12.83)	Fair
**Job strain**						
Aberg et al 2022 (65)	Sweden	Cohort	General working population (men)	1 483 310	HR (95% CI) for active work 0.54(0.48–0.62), high strain work 0.99 (0.86–1.15), passive work 1.17 (1.04–1.31)	Good
Almroth et al 2022 (62)	Sweden	Cohort	General working population	3 017 962	HR (95% CI) for job strain; men and women respectively: 1.13 (1.04–1.24) and 1.14 (0.99–1.33)	Good
Baumert et al 2014 (33)	Germany	Cohort	General population	6817	HR (95% CI) for job strain 0.99 (0.45–2.20)	Good
Niedhammer et al 2021 (29)	France	Cohort	General working population	1 496 332	HR (95% CI) for job strain men and women respectively: 1.28 (1.09–1.51) and 1.35 (1.03–1.77)	Good
Pan et al 2022 (28)	Sweden	Cohort	General working population	4 458 673	HR (95% CI) for active work; men and women respectively: 0.77 (0.71–0.84) and 0.86 (0.75–0.98). HR (95% CI) for passive work; men and women respectively: 1.19 (1.12–1.27) and 1.38 (1.23–1.54). HR (95% CI) for high strain work; men and women respectively: 1.04 (0.94–1.16) and 1.04 (0.84–1.30).	Good
**Social support**						
Dempsey et al 2021 (66)	US	Case–control	Specific sector/occupation	187–189	OR (95% CI) based on supervisor or next of kin reports respectively: Could rely on other members in his/her unit 0.8 (0.4–1.6) and 1.0 (0.5–1.9), thought the members of his/her unit knew they could depend on each other 1.7 (0.9–3.1) and 0.9 (0.5–1.8), thought the members of his/her unit stood up for each other 1.2 (0.7–2.2) and 0.9 (0.5–1.7)	Poor
Niedhammer et al 2021 (29)	France	Cohort	General working population	1 496 332	HR (95% CI) for low support men and women respectively: 1.19 (1.00–1.42) and 1.27 (0.94–1.71). HR (95% CI) for isostrain (job strain and low support); men and women respectively: 1.29 (1.09–1.53) and 1.36 (1.04–1.78).	Good
Schneider et al 2011 (36)	Germany	Case–control	General population	559	OR (95% CI) for lower levels of satisfaction with superiors 1.1 (0.8–1.5), lower levels of satisfaction with colleagues 1.2 (0.8–1.99)	Poor
**Workplace violence**						
Magnusson Hanson et al 2023 (12)	Sweden, Finland, Denmark	Cohort	General working population	205 048	HR (95% CI) for workplace violence 1.76 (1.21–2.54)	Good
**Workplace bullying**						
Conway et al 2022 (31)	Denmark	Cohort	General working population	98 330	HR (95% CI) for workplace bullying 2.21 (0.87–5.64) ^b^	Good
Magnusson Hanson et al 2023 (12)	Sweden, Finland, Denmark	Cohort	General working population	191 783	HR (95% CI) for workplace bullying 1.59 (0.97–2.62)	Good
**Workplace sexual/gender based harassment**						
Magnusson Hanson et al 2020 (32)	Sweden	Cohort	General working population	85 205	HR (95% CI) for workplace sexual harassment 2.82 (1.49–5.34)	Good
**Other psychosocial factors**						
Baumert et al 2014 (33)	Germany	Cohort	General population	6817	HR (95% CI) for psychosocial working conditions 1.63 (0.75–3.53) covering disturbances and interruptions, urge for fast decisions, high responsibility for people, high responsibility for machines, strong competition	Good
Dempsey et al 2021 (66)	US	Case–control	Specific sector/occupation	187–189	OR (95% CI) based on supervisor or next of kin reports respectively: Trusted his/her leaders 0.6 (0.3–1.1) and 0.7 (0.4–1.3), thought the members of his/her unit were cooperative 1.3 (0.7–2.2) and 1.0 (0.5–1.9)	Poor
Feskanisch et al 2002 (37)	US	Cohort	Specific sector/occupation (women)	94 110	RR (95% CI) for minimal work stress 2.4 (0.9–6.1) moderate work stress 1.1 (0.5–2.5), severe work stress 1.9 (0.8–4.7)	Fair
Schneider et al 2011 (36)	Germany	Case–control	General population	559	OR for for psychic strain through contact with clients 1.1 (0.7–1.6)	Poor

^a^ Law et al examined work–related life events (such as a new job, job changes, had been fired, in danger of losing job or being demoted, had conflicts with boss or colleagues, had resigned, or had had a pay cut or pay freeze) labelled “job insecurity” since a high proportion of cases were in danger of losing their job or being demoted. ^b^ HR 1.78 (95% CI 1.16–2.73) was found when results were pooled with those of Conway et al 2022.

**Table 2:** Summary of studies in the review addressing associations between employment/working conditions and suicide attempt, including their main characteristics, presented risk estimates adjusted for covariates, and overall study quality (according to NOS).

Exposure and study	Country	Study design	Study sample	Sample size	Risk estimates	Quality
**Shift work**						
Armstrong et al 2023 (67)	Australia	Cohort	General population (men)	1564	OR (95% CI) for night work 1.75 (1.05–2.91)	Poor
**Precarious work**						
Jonsson et al 2021 (35)	Sweden	Cohort	General population	2 743 764	HR (95% CI) for constant precarious employment trajectory; all, men and women respectively: 1.22 (1.14–1.31) and 1.28 (1.17–1.41) and 1.14 (1.02–1.28) HR (95% CI) for constant low quality employment trajectory; all, men and women respectively: 1.30 (1.11–1.52) and 1.45 (1.22–1.71) and 1.02 (0.77–1.35) HR for constant business owner employment trajectory; all, men and women respectively: HR (95% CI) for solo self–employment trajectory; all, men and women respectively: 0.95 (0.86–1.04), 0.97 (0.87–1.07), 0.99 (0.83–1.19). Results for unemployment and changing trajectories and/mixed trajectories not reported here	Good
**Job loss**						
Milner et al 2014 (58)	Australia	Case–control	General population	340	OR (95% CI) for job loss 1.67 (0.76–3.65)	Good
**Job insecurity**						
Blomqvist et al 2022 (60)	Sweden	Cohort	General working population	65 571	HR (95% CI) for job insecurity; all, men and women respectively: 1.03 (0.86–1.24) and 1.20 (0.93–1.54) and 0.88 (0.68–1.14)	Good
**Job demands**						
Almroth et al 2022 (62)	Sweden	Cohort	General working population	3 017 962	HR (95% CI) for high demands men and women respectively: 0.88 (0.81–0.95) and 0.86 (0.80–0.94)	Good
Ostry et al 2007 (30)	Canada	Case–control	Specific sector/occupation	508	OR (95% CI) for higher levels of demands 0.98 (0.87–1.10)	Fair
**Job control**						
Almroth et al 2022 (62)	Sweden	Cohort	General working population	3 017 962	HR (95% CI) for low job control; men and women respectively: 1.51 (1.37–1.67) and 1.43 (1.32–1.56)	Good
**Job strain**						
Almroth et al 2022 (62)	Sweden	Cohort	General working population	3 017 962	HR (95% CI) for job strain; men and women respectively: 1.09 (1.02–1.16) and 1.04 (0.97–1.10)	Good
**Social support**						
Ostry et al 2007 (30)	Canada	Case–control	Specific sector/occupation	508	OR (95% CI) for higher social support 0.73 (0.54–0.98)	Fair
Padmanathan et al 2023 (68)	UK	Cohort	Specific sector/occupation	22 501	OR (95% CI) for lack of managerial support; clinical and non–clinical workers respectively: 0.50 (0.18–1.37) and 1.22 (0.55–2.73)	Poor
**Workplace violence**						
Magnusson Hanson et al (12)	Sweden, Finland, Denmark ^a^	Cohort	General working population	146 301	HR (95% CI) for workplace violence 1.26 (1.06–1.49)	Good
**Workplace bullying**						
Conway et al 2022 (31)	Denmark	Cohort	General working population	98 330	HR (95% CI) workplace bullying 1.77 (1.19–2.63)	Good
Magnusson Hanson et al 2023 (12)	Sweden, Finland, Denmarkb	Cohort	General working population	93 453	HR workplace bullying 1.21 (0.96–1.52) ^b^	Good
**Workplace sexual/gender based harassment**						
Magnusson Hanson et al 2020 (32)	Sweden	Cohort	General working population	85 205	HR for workplace sexual harassment 1.59 (1.21–2.08)	Good
**Other psychosocial factors**						
Padmanathan et al 2023 (68)	UK	Cohort	Specific sector/occupation	22 501	OR for exposure to potentially morally injurious events; clinical and non–clinical workers respectively: 0.84 (0.33–2.16) and 1.30 (0.60–2.84)	Poor

^a^ Data from Denmark not used for separate analyses on suicide or suicide attempt ^b^ HR 1.33 (95% CI 1.09–1.62) was found when results were pooled with those of Conway et al 2022.

### Results of studies on working conditions and suicide

**Figure 2:**
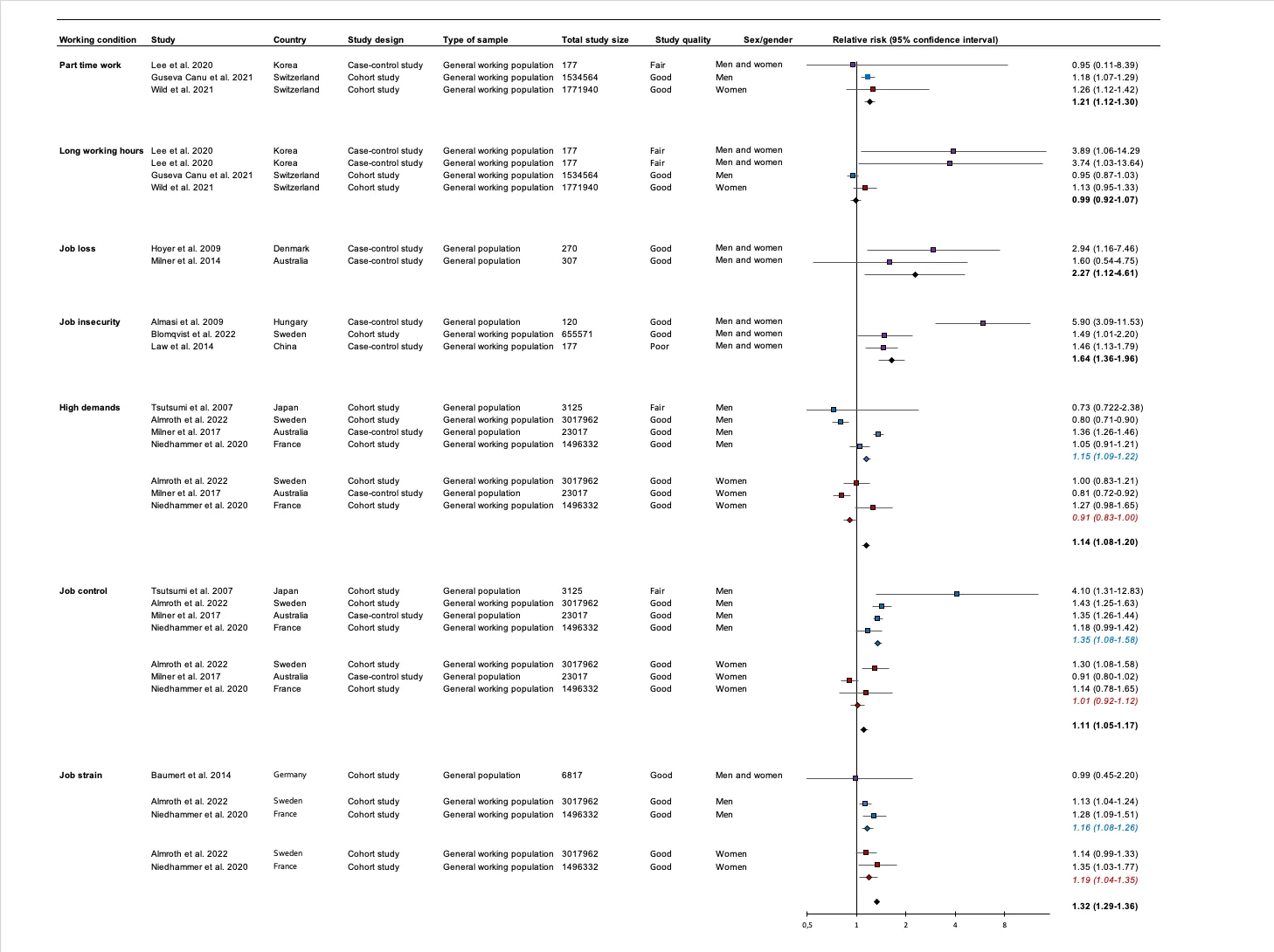
Results from meta-analyses on employment/working conditions and suicide and when possible sub-group analyses for men and women. The overall pooled relative risk estimates are presented in bold and the pooled estimated risk estimates for men and women separately are presented in blue (men) or red (women) and italic. The Figure also shows the separate risk estimates in the meta-analysis, the main characteristics of the underlying original studies and whether estimates concerned men and women (purple), men only (blue) or women only (red).

Figure 2 summarizes the results on employment/working conditions and suicide for the studies included in the meta-analysis. We performed meta-analyses for part-time work, long working hours, job loss, job insecurity, high job demands, low job control and job strain. Table 1 presents the results from all studies on suicide, including those not eligible for inclusion in the meta-analyses.

### Employment or working conditions and studies included in meta-analyses

The meta-analysis on part-time work showed a pooled RR of 1.21 (95% CI 1.12–1.30, N=3). No association was found for long working hours of ≥45 hours per week (pooled RR 0.99, 95% CI 0.92–1.07). The latter analysis was based on the same three studies reporting estimates of various forms of long working hours (≥40 hours per week), and the result was unaltered in a sensitivity analysis only including independent estimates, which excluded one of the estimates from Lee et al (>52 h/w). Analysis on 2 of 3 job loss studies (the 2 studies that presented risk estimates), resulted in a pooled RR of 2.27 (95% CI 1.12–4.62). Moreover, job insecurity was consistently connected to suicide (pooled risk estimate 2.10 (95% CI 1.50–2.94, N=3). Further, a meta-analysis on high job demands showed a summary risk estimate of 1.14 (95% CI 1.08–1.20, based on 4 of the 7 studies on demands). Subgroup analyses on sex-stratified results or studies restricted to male or female participants further indicated an association with high demands for men (RR 1.15, 95% CI 1.08–1.21), but not women (RR 0.91, 95% CI 0.83–1.00). However, separate analysis of the 2 studies that analyzed demands on a continuous scale, indicated a lower risk for suicide following higher demands (RR 0.88, 95% CI 0.80–0.98). Similarly, an analysis on low job control and suicide, based on the same 4 studies, yielded a summary estimate of 1.11 (95% CI 1.05–1.17), and again subgroup analyses were indicative of an association among men (RR 1.35, 95% CI 1.28–1.43), but not among women (RR 1.01, 95% CI 0.92–1.12). Finally, job strain was connected to suicide [summary RR 1.32 (95% CI 1.29–1.36), N=3].

### Employment or working conditions and studies not included in meta-analyses

Other studies on job demands and suicide, analyzing for example increasing or decreasing demands or trajectories of demands, presented mixed findings. Low job control trajectories, though, were associated with suicide for both men and women in one paper (28). Social support at work was examined in two studies suggesting an increased risk of suicide following low social support and decreased risk following increasing support (29, 30). These two studies were considered separately due to the heterogeneity in exposure assessment. Furthermore, two studies had examined exposure to workplace bullying (12, 31), and the previously reported meta-analytic estimate from these studies were RR 1.78 (95% CI 1.16–2.73). Similar findings were shown by Magnusson Hanson et al (12) regarding exposure to violence (HR 1.76, 95% CI 1.21–2.54), and exposure to workplace sexual harassment (HR 2.82, 95% CI 1.49–5.34) (32). Additionally, we identified three studies on shift work of varying quality of which two found an association between some form of shift work and suicide (33, 34), although one examined shift work, and night work together with overtime-, task- and assembly line work (33). Further, we found two studies of varying quality focusing on types of employment usually considered as precarious/atypical (35, 36), which showed mixed results. There was also one study (33) focusing on psychosocial working conditions in general, showing a tendency towards increased risk for suicide (HR 1.63, 95% CI 0.75–3.53). Finally, one study (37) examined general ratings of stress at work, and found tendencies towards an association with suicide (RR 1.9, 95% CI 0.9–4.7 for severe work stress) and indicated that combined home and work stress increased the risk for suicide (RR 4.9, 95% CI CI 1.4–17). However, no studies were identified that addressed effort–reward imbalance, workplace justice or workplace conflicts.

### Results of studies on employment or working conditions and suicide attempt

The results from all studies on suicide attempt are summarized in table 2. In single studies, we noted statistically significant associations for night shift work, precarious employment trajectories, low job control, job strain (men), social support, workplace violence, bullying and sexual harassment, while risk estimates for suicide attempts among exposed to job loss, job insecurity, and high/higher job demands were not statistically significantly different for those of unexposed. No meta-analysis on suicide attempt was conducted due to heterogeneity in exposures, design or outcomes. However, a meta-analytic estimate for workplace bullying and suicide attempt was published in a previous review (12), which was RR 1.33 (95% CI 1.09–1.62) based on data from 11 different studies altogether (2 analyzed separately and 9 analyzed through a pooled dataset) (12, 31). No studies were identified that addressed part-time work, long working hours, effort–reward imbalance, workplace justice or workplace conflicts.

### Assessment of certainty of evidence

Because the evidence was based on observational studies, our GRADE based quality of evidence assessment started with “low” (27). Regarding suicide, we upgraded exposure to job loss to “moderate” quality of evidence because of large effect size. Exposure to job insecurity was downgraded due to indirectness and upgraded due to large effect size, yielding an assessment of “low” quality of evidence. All other exposures (high job demands, low job control, part-time work, long working hours, job strain and workplace bullying) were downgraded for different reasons (see table 3 for details), yielding a quality assessment of “very low” for each of these exposures. For the remaining exposures (workplace violence, workplace sexual harassment, shift work, low social support, precarious employment), assessment of quality of evidence was not performed because of lack of findings from multiple studies. With regard to suicide attempt, we could only conduct a quality of evidence assessment for workplace bullying, for which we downgraded the quality of evidence to “very low” due to inconsistency (table 3 for details). For the remaining exposures (workplace violence, workplace sexual harassment, job demands, social support, job strain, shift work, job loss, precarious employment, job insecurity, low job control, part-time work, long working hours), assessment of quality of evidence was not performed, because of lack of findings from multiple studies.

**Table 3:** Assessment of GRADE. [HR=hazard ratio; OR=odds ratio; RR=relative risk.]

Outcome and exposure	Relative effects from meta-analysis/other studies	Participants in meta-analysis / other studies (N)	Studies in meta-analysis / other studies (N)	Certainty of evidence (GRADE)	Comments
**Suicide**					
**Exposures assessed in ≥5 studies**					
High job demands	RR (95% CI) 1.14 (1.08–1.20)/ OR (95% CI) 0.9 (0.7–1.2), OR (95% CI) 1.4 (1.1–1.8), OR (95% CI) 0.78 (0.69–0.89), men and women respectively: medium–low trajectory: HR (95% CI) 0.81 (0.76–0.87) and HR (95% CI) 1.08 (0.93–1.26), medium–high trajectory HR (95% CI) 0.72 (0.66–0.78) and HR (95% CI) 0.87 (0.73–1.05), high trajectory HR (95% CI) 0.76 (0.67–0.86) and HR (95% CI) 1.03 (0.81–1.33)	9 000 316 / 4 540 995	5/2	Very low. Downgrading due to inconsistency and indirectness	In 3 of the 4 studies in the meta–analysis, presenting sex–stratified results, the exposure assessment was based on a job-exposure matrix. In a separate meta-analysis higher job demands were associated with a lower risk estimate for suicide. However, while most of the studies assessed aspects from job demand–control model such as working fast, working hard, excessive workload/efforts, having enough time and conflicting demands, Schneider assessed time pressure and responsibility.
Low job control	RR (95% CI) 1.13 (1.07–1.18)/no results presented, men and women respectively: medium–high trajectory HR (95% CI) 1.35 (1.24–1.47) and HR (95% CI) 1.20 (1.04–1.39), medium–low trajectory HR (95% CI) 1.49 (1.36–1.63) and HR (95% CI) 1.54 (1.32–1.80), low trajectory HR (95% CI) 1.49 (1.33–1.68) and HR (95% CI) 1.32 (1.10–1.60)	9 000 316 / 4 540 995	5/2	Very low. Downgrading due to indirectness	In 3 of the 4 studies in the meta-analysis, presenting sex-stratified results, the exposure assessment was based on a job-exposure matrix. Furthermore, while most of these studies assessed aspects from job demand-control model such as having freedom to decide how and when the work should be done, and possibility to use expertise, creativity, and learning new things in the job, Schneider et al (2011) only investigated monotonous work.
**Exposures assessed in <5 studies**					
Part-time work	RR (95% CI) 1.21 (1.12–1.42) / -	3 320 988 / -	3 / -	Very low. Downgrading due to inconsistency	
Long working hours	RR (95% CI) 0.99 (0.95–1.33) / -	3 320 988 / -	3 / -	Very low. Downgrading due to imprecision	There is also some inconsistency and probable indirectness, and 2 of the studies are from the same source population (one on men and one on women)
Job insecurity	RR (95% CI) 2.10 (1.13–1.79) / -	65 868 / -	3 / -	Low. Downgrading due to indirectness and upgrading due to large effect size	Job insecurity had been operationalized in different ways. Law et al (2014) measured work-related life events (such as a new job, job changes, had been fired, in danger of losing job or being demoted, had conflicts with boss or colleagues, had resigned, or had had a pay cut or pay freeze) but labelled their variable job insecurity since it was common to be in danger of losing job or being demoted. Blomqvist et al (2022) examined threat of dismissal and Almasi et al (2009) studied concerns over work prospects.
Job strain	RR (95% CI) 1.32 (1.29–1.36) / -	4 521 111 / -	3 / -	Very low. Downgrading due to indirectness	Some studies instead analyzed situations with low strain, passive work, active work or high strain, and with different comparison groups, thus not considered comparable. One study provided an estimate for high strain of HR (95% CI) 0.99 (0.86–1.15) compared to low strain, and another study estimates for high strain of HR (95% CI) 1.41 (1.18–1.68) for men and HR (95% CI) 1.31 (0.94–1.83) for women) compared to active work. Estimates of trajectories of high strain work were HR (95% CI) 1.04 (0.94–1.16) for men and HR (95% CI) 1.04 (0.84–1.30) for women.
Job loss	RR (95% CI) 2.27 (1.12–4.62)/ ^a^ P<0.001	1215/577	2/1	Moderate. Upgrading due to large effect size	Analyses of suicide or suicide attempt was also suggestive of an association in Milner et al 2014
Workplace bullying	RR (95% CI) 1.78 (1.16–2.73) ^b^	290 113/–	2 / -	Very low. Sowngrading due to inconsistency	
Workplace violence	RR (95% CI) 1.76 (1.21–2.54)	- / 146 301	- / 1	-	Only 1 study reported results for workplace violence
Workplace sexual harassment	RR (95% CI) 2.82 (1.49–5.34)	- / 84 238	- / 1	-	Only 1 study reported results for workplace sexual harassment
Shift work	- / shift work daytime for men HR (95% CI) 1.39 (1.19–1.63) and women HR (95% CI) 1.06 (0.78–1.44), Shift work nighttime for men HR (95% CI) 0.97 (0.81–1.16) and women HR (95% CI) 0.95 (0.42–2.14) Shift work daytime and nighttime for men HR (95% CI) 1.38 (1.19–1.61) and women HR (95% CI) 1.98 (0.28–14.14)	- / 1 511 456	- / 1	-	Only 1 study reported results for different types of shift work, while another reported results for chronobiological exposures that could include also overtime work, taskwork, and/or assembly–line work. The risk estimate for chronobiological exposure was HR (95% CI) 2.32 (1.01–5.35).
Low social support	- / low social support for men HR (95% CI) 1.18 (0.99–1.42) and women HR (95% CI) 1.27 (0.94–1.71)	- / 1 496 332	- / 1	-	The same study also reported results for isotrain (i.e. job strain and low social support); for men HR (95% CI) 1.29 (1.09–1.53) and for women HR (95% CI) 1.36 (1.04–1.78)
Precarious employment	- / temporary employment 2.3 (0.8–6.8)	- / 559	- / 1	-	Only 1 study reported results on an employment form that could be considered precarious
**Suicide attempt**					
**Exposures assessed in < 5 studies**					
Workplace bullying	RR (95% CI) 1.33 (1.09–1.62) ^b^ / -	191 783 / -	2 / -	Very low downgrading due to inconsistency	
Workplace violence	- / 1.26 (1.06–1.49)	- / 146 301	- / 1	-	
Workplace sexual harassment	- / 1.59 (1.21–2.08)	- / 83 600	- / 1	-	
Job demands	- / high demands for men HR (95% CI) 0.88 (0.81–0.95) and women HR (95% CI) 0.86 (0.80–0.94), higher demands OR (95% CI) 0.98 (0.87–1.10)	- / 3 018 470	- / 2	-	2 studies reported results, but only one assessed high job demands
Social support	- / increasing social support OR (95% CI) 0.73 (0.54–0.98), lack of managerial support OR (95% CI) 0.50 (0.18–1.37) in clinical and OR (95% CI) 1.22 (0.55–2.73) in non–clinical workers	- / 23 009	- / 2	-	Only 1 study reported results measured according to the demand-control-support model
Job strain	- / for men HR (95% CI) 1.09 (1.02–1.16) and for women HR (95% CI) 1.04 (0.97–1.10)	- / 3 017 962	- / 1	-	Only 1 study reported results for job strain, while another reported results for situations with low strain, passive work, active work or high strain. The risk estimate for high strain was HR (95% CI) 1.11 (1.03–1.21) compared to low strain.
Shift work	- / night work OR (95% CI) 1.75 (1.05–2.91)	- / 1564	- / 1	-	Only 1 study reported results for some type of shift work
Job loss	- / OR (95% CI) 1.67 (0.76–3.65)	- / 340	- / 1	-	
Precarious employment	- / precarious employment trajectory HR (95% CI) 1.22 (1.14–1.31)	- / 2 743 764	- / 1		
Job insecurity	- / HR (95% CI) 1.03 (0.86–1.24)	- / 65 571	- / 1	-	
Low job control	- / for men HR (95% CI) 1.51 (1.37–1.67) and for women HR (95% CI) 1.43 (1.32–1.56)	- / 3 017 962	- / 1	-	
Part-time work	- / -	- / -		-	
Long working hours	- / -	- / -		-	

^a^ Risk estimate not presented in the study. ^b^ Reported in Magnusson Hanson et al 2023 (12).

## Discussion

### Summary of results

This systematic review of 27 longitudinal studies on employment or working conditions and suicidal behaviors included data from a variety of countries, study populations, and a wide range of work-related factors. Our meta-analyses yielded statistically significant associations with suicide for the following conditions: part-time work, job loss, job insecurity, high (or increasing) job demands, low job control, job strain and workplace bullying. We assessed certainty of evidence as “moderate” for job loss and as “low” for job insecurity. For all other conditions, we assessed certainty of evidence as “very low”, meaning that the true effect estimate for suicide might be substantially different from the estimates that we calculated in the meta-analyses. The number of studies on employment/working conditions and suicide attempts were more limited and we could assess certainty of evidence only for an association between workplace bullying and suicide attempt, which we assessed as very low. For all other conditions, we were unable to estimate whether they were associated with risk of suicide attempt and therefore unable to assess the certainty of evidence.

### General interpretation of results in the context of other evidence

Certainty of evidence was highest for job loss (moderate certainty of evidence) and job insecurity (low certainty of evidence), which is in agreement with previous research on the role of unemployment in suicide risk (38–41) and job insecurity in suicidal ideation (8). To the best of our knowledge, there has been only one systematic review before, by Milner et al (8), examining a number of different working conditions and suicidal behavior. Whereas their review was mainly based on cross-sectional studies and studies on suicidal ideation – and only included 5 longitudinal studies on suicide or suicide attempt – our study included a total of 27 longitudinal studies on these two outcomes. Our review also covered a substantially larger number of work-related factors than Milner et al's review, reflecting the growing number of longitudinal studies on employment/working conditions and suicidal behavior in recent years. Milner et al's review indicated that low job control was associated with an increased risk of suicide and our results corroborated this finding. Moreover, we also found an association for job demands and the combination of high job demands and low job control, denoted as job strain in the literature, and risk of suicide.

Previously Leach et al (9) noted one cross-sectional study in the literature on the association between workplace bullying and risk of suicide attempts, and a prospective association with both suicide attempt and suicide was supported in our earlier work (12, 31) and the present review. Moreover, our review suggests that exposures to several other types of offensive behaviors, such as workplace violence or workplace sexual harassment might increase the risk of suicide, although this conclusion is limited by the low number of studies conducted on these exposures.

Extreme working hours have also been suspected to increase suicide risk and in the Japanese language/culture the term karo-jisatsu (suicide by overwork) is used for such a potential relationship (42). However, we found the evidence generally insufficient for drawing conclusions about associations between working time arrangements and suicidal behaviors. Although we identified several studies on long working hours, the operationalization differed between studies, and none of the studies had investigated ≥55 hours per week (which is commonly compared to standard working hours (35–40 hours per week) in the research literature). Moreover, the studies by Guseva-Canu (34) and Wild (43) are likely to have assessed contractual working hours rather than actual hours (including overtime). More high-quality research is therefore needed on long working hours and suicidal behaviors, also considering some previous work suggesting an association between long working hours and suicidal ideation (44) and with mental health problems (45). The situation was also similar for precarious employment, although we found a statistically significant association between part-time work and suicide, which could be associated with precarious employment conditions such as irregular hours, poor pay, and other insecurities. This calls for further research on temporary employment and other forms of precarious employment and suicidal behaviors.

Employment or working conditions such as job loss, job insecurity, high job demands, low job control, job strain and workplace bullying may influence health-related behaviors (eg, smoking, risky alcohol consumption), that in turn could lead to various health-related outcomes (46). Adverse employment/working conditions may also be associated with prolonged stress responses and bodily changes leading to diseases including diabetes, cardiovascular disease and depression (46–48). With regard to depression, depression and other mental disorders are established risk factors for suicidal behavior (1), and there is a growing research literature suggesting that some adverse working conditions are associated with an increased risk of depressive disorder (49, 50), including working conditions, such as high job strain, low job control and high job insecurity; factors that were also associated with an increased risk of suicidal behaviors in our review.

### Strengths and limitations of the review

We focused on observational studies including cohort and case–control studies with a prospective or retrospective design that followed workers over a period of time with regard to outcome and/or exposure. These types of studies provide stronger evidence than, eg, cross-sectional studies and case series/reports, largely because the potential cause is assessed before the potential effect/outcome which increases the chances of identifying a causal effect. Unfortunately, we identified no intervention study in our literature search. Such studies would have contributed to stronger evidence regarding causality. We followed a pre-published protocol, and our search strategy is likely to cover a broad range of literature on the topic. We also used explicit eligibility criteria and carried out a risk of bias assessment by means of the NOS, which is considered useful for reviews of non-randomized studies and has been used earlier by the research team (51). A potential limitation is that we only searched in commonly used databases, and we therefore might have missed some studies that had been indexed in more rarely used databases. Further, we decided to only include published, peer-reviewed articles to ensure a further quality-control of the work, which might have caused us to miss a few studies. Other possible limitations of the search strategy were that the first screening was based on abstracts, title or keywords and we might have missed articles that indicated employment/working conditions or the outcome suicidal behavior in the main text only.

We carried out meta-analyses to improve the interpretation of the results by increasing the precision in estimates of risk and decreasing the risk of missing an association due to low precision in separate studies. The meta-analyses were, however, mostly based on few studies, and the studies were often heterogeneous in terms of design, samples and size. The findings should thus be interpreted cautiously and may be influenced by specific historic or cultural characteristics resulting in extreme findings in single studies. Because of the small number of studies, we were also restricted to fixed-effects meta-analysis based on an assumption that all effect estimates are estimating the same underlying effect which may not be fulfilled (52). Further, because of the low number of studies in the meta-analyses, we could not conduct funnel plots to examine possible publication bias (25), and consequently we cannot rule out that publication bias has affected our estimates.

As in all reviews, the results of our review must be interpreted in consideration of the limitations of the primary studies. In contrast to the previous meta-analysis on work characteristics and suicidality (8), which was based on crude estimates, we summarized results from models adjusted for sociodemographic characteristics such as sex, age, and education when available. Some of the work also adjusted for additional factors such as economic situation, life tyle factors, or prior health. However, only a few studies considered other potential work-related risk factors, and non-work-related risk factors such as divorce and other life events, and therefore we cannot exclude that residual confounding affects our estimates. Another limitation is that few or none of the studies addressed factors such as workplace social support, effort–reward imbalance, and workplace justice and few looked at suicide attempt. Moreover, several studies had limitations with regard to ascertainment of exposure, as they were based on self-reports, which carry a risk of information bias and in some cases even recall bias risking to overestimate associations. Another limitation of the existing evidence base is that the number of studies concerning each of the examined psychosocial working conditions was to small to examine the role of cultural variation in effects. The impact of psychosocial working conditions may be shaped by the social and cultural context, but such effect modification could not be assessed given the limited number of studies. Some studies were based on job exposure matrices with lower risk of information bias but higher risk of non-differential misclassification of exposure. In some studies, selection bias may have been an issue as there were differences in selection of cases and selection of controls. Moreover, it is possible that certain groups of individuals, with increased risk for mental distress or suicidality, might have been employed in sectors or occupations with increased risk for certain adverse working conditions (53). We also noted that some of the studies on suicide attempts failed to demonstrate that participants did not have suicide attempts in the past, in which case we cannot be certain that the exposure preceded the outcome.

### Concluding remarks

This systematic review of 27 longitudinal studies identified seven working conditions that were associated with an increased risk of suicide, but found that the certainty of evidence for the associations was mostly very low. The certainty of evidence was assessed as higher for two of these conditions, job loss (moderate) and job insecurity (low), but pooled risk estimates in our meta-analyses were based on only a few studies on the topics. For several of the employment/working conditions certainty of evidence was, however, not possible to assess because of the lack of findings from multiple studies. With regard to suicide attempt, we could conduct a certainty of evidence assessment only for workplace bullying, and deemed the certainty of evidence to be very low. We conclude that in recent years, an increasing number of studies suggest that working conditions may contribute to the etiology of suicidal behavior. However, the research field is still in its early stages. Further research is needed on associations between working conditions and suicide attempts to understand links between working conditions beyond job loss and suicide, and which address methodological limitations in the existing literature for more certain evidence.

## Supplementary material

Supplementary material
