# Supplementary material for: Employment and working conditions and risk of suicidal behaviors: a systematic review and meta-analysis of longitudinal studies
Source: Scand J Work Environ Health. 2026 Aug 31;52(5):486–501. doi: 10.5271/sjweh.4308 (PMC13541624; doi:10.5271/sjweh.4308)
Supplement: Supplementary file 1 [file SJWEH-52-486-S001.pdf]

Employment and working conditions and risk of suicidal behaviors: a systematic review and meta-analysis of longitudinal studies[1]

by Linda L Magnusson Hanson, PhD,**[2] **Ida EH Madsen, PhD, Sandra Blomqvist, PhD, Rebecka Holmgren, PhD, Kathrine Sørensen, PhD, Reiner Rugulies, PhD

1. Supplementary material
2. Correspondence to: Linda Magnusson Hanson, Division of Psychobiology and Epidemiology, Department of Psychology, Stockholm University, Sweden. [E-mail: linda.hanson@su.se]

Appendix 1. Search strategy and list of search terms

The following databases were searched for keywords in abstracts or title/abstract/keywords: Embase, PsycInfo, Scopus, and Web of Science. Web of Science citation indexes or databases included Web of Science Core Collection, MEDLINE, KCI-Korean Journal Database, SciELO Citation Index, and Russian Science Citation Index. The search was refined by Year: 1995-, and Language: English. The search strategy was initially developed in collaboration with librarians at Lund University for a review performed for the Swedish Agency for Work Environment Expertise.

**Search terms**
S1: work* OR job* OR employ* OR leader* OR manager* OR supervis* OR boss* OR team* OR organizational* OR occupational*
S2: suicid* OR parasuicid*

S3: cohort* OR prospective OR longitudinal OR “follow-up stud*” OR “incidence stud*” OR case-control OR “retrospective mortality” OR intervention OR “randomized controlled” OR review OR meta-analysis

Data extraction

We extracted the following information from the included studies: author, year of publication, country, study design, study population, sample size, recruitment strategy, exposures, outcomes, statistical method, covariates used in analyses, and risk estimates for exposures in focus for the review

If several risk estimates were presented, we focused on the estimate from a model that included the largest number of confounders considered important at the outset such as sex, age, and socioeconomic status/education.

Study quality

The Newcastle-Ottawa Scale (NOS) consists of separate scales for cohort and case-control studies covering 3 domains: selection of study groups (max 4 stars), comparability of the groups (max 2 stars), and ascertainment of exposure or outcome (max 3 stars). For each item in the scales, a star is used to indicate high quality, with the exception for the item on comparability for which a study can be rewarded with 2 stars. Consequently, the maximum numbers of stars were 9.

Supplementary Table S1. Characteristics of the studies included in the systematic review

**Author**	**Year**	**Country**	**Study design**	**Sample (specific sex/gender)**	**Sample size**	**Exposure**	**Outcome**
Aberg et al. (65)	2022	Sweden	Cohort	General working population (men)	1483310	High strain-, active-, passive-, or low strain (ref) work	Suicide, suicide or suicide attempts
Almasi et al. (59)	2009	Hungary	Case-control	General population	85 cases/35 controls	Concern over work prospects	Suicide
Almroth et al. (62)	2022	Sweden	Cohort	General working population	3017962	Job demands, job control, job strain	Suicide, suicide attempts
Armstrong et al. (67)	2023	Australia	Cohort	General population	1564	Night work	Suicide attempt
Baumert et al. (33)	2014	Germany	Cohort	General population	6817	psychosocial working conditions (disturbances and interruptions, urge for fast decisions, high responsibility for people, high responsibility for machines, strong competition), job strain, chronobiological exposures (overtime, shift work, night shifts,	Suicide

						taskwork, assembly-line work)	
Blomqvist et al. (60)	2022	Sweden	Cohort	General working population	65571	Job insecurity	Suicide, Suicide attempt
Conway et al. (31)	2022	Denmark	Cohort	General working population	98330	Workplace bullying	Suicide, suicide attempt
Dempsey et al. (66)	2021	US	Case-control	Specific sector/occupation	107 cases/80 controls (supervisor analysis), 61 cases/128 controls (next of kin analysis)	Unit cohesion	Suicide
Feskanisch et al. (37)	2002	US	Cohort	Specific sector/occupation (women)	94110	Work stress	Suicide
Guseva Canu et al. (34)	2021	Switzerland	Cohort	General working population (men)	1534564	Working hours	Suicide
Hoyer et al. (56)	2009	Denmark	Case-control	General population	135 cases/135 controls	Job loss	Suicide
Jonsson et al. (35)	2021	Sweden	Cohort	General population	2743764	Low quality employment trajectories	Suicide attempt
Kolves et al. (57)	2006	Estonia, Germany	Case-control	General population	156 cases/156	Job loss	Suicide

					controls Estonia, 163 cases/163 controls Germany		
Law et al. (61)	2014	China	Case-control	General working population	63 cases/114 controls	Work-related life events (such as a new job, job changes, had been fired, in danger of losing job or being demoted, had conflicts with boss or colleagues, had resigned, or had had a pay cut or pay freeze) labelled "job insecurity" since a high proportion of cases were in danger of losing their job or being demoted	Suicide
Lee et al. (54)	2020	Korea	Cohort	General working population	14484	Working hours	Suicide
Magnusson Hanson et al. (32)	2020	Sweden	Cohort	General working population	85205	Workplace sexual harassment	Suicide, suicide attempt
Magnusson Hanson et al. (12)	2023	Sweden, Finland, Denmark	Cohort	General working population	205048 (violence) or 191783	Workplace violence, workplace bullying	Suicide, suicide attempt

					(bullying, 93453 in previously unpublished data)		
Milner et al. (63)	2017	Australia	Case-control	General population	9010 cases/ 14007 controls	Job demands, job control	Suicide
Milner et al. (58)	2014	Australia	Case-control	General population	84 cases suicide/223 controls, and 101 cases suicide attempt/239 controls	Job dismissal or redundancy referred to as involuntary job loss	Suicide, suicide attempt
Niedhammer et al. (48)	2021	France	Cohort	General working population	1496332	Job demands, job control, job strain, low strain-, active- (ref), passive, high strain work, workplace social support, isostrain	Suicide
Niedhammer et al. (53)	2022	France	Cohort	General working population	1511456	Shift work, night work	Suicide
Ostry et al. (30)	2007	Canada	Case-control	Specific sector/occupation (men)	162 cases suicide/486 controls, 127 cases suicide	Job demands, job control, workplace social support	Suicide, suicide attempt

					attempt/381 controls		
Padmanathan et al. (68)	2023	UK	Cohort	Specific sector/occupation	22501	Managerial support, exposure to potentially morally injurious events	Suicide attempt
Pan et al. (28)	2023	Sweden	Cohort	General working population	4458673	Job demands, job control	Suicide
Schneider et al. (36)	2011	Germany	Case-control	General population	163 cases/396 controls	Temporary employment, self-employment, monotonous work, responsibility, time pressure, satisfaction with colleagues/superiors, psychic strain through contact with clients	Suicide
Tsutsumi et al. (64)	2007	Japan	Cohort	General population (men)	3125	Job demands, job control	Suicide
Wild et al. (55)	2021	Switzerland	Cohort	General working population (women)	1771940	Working hours	Suicide

Supplementary Table S2. Risk of bias assessment according to the Newcastle-Ottawa Scale (NOS)

Author	Year	Country	Study design	NOS domain selection	NOS domain selection	NOS domain selection	NOS domain selection	NOS don comparal
				Is the case definition adequate? / Representativeness of exposed cohort	Representativeness of cases/ Selection of non-exposed cohort	Selection of controls/ Ascertainment of exposure	Definition of controls/ Demonstration that outcome of interest was not present at start of the study	Comparab of cases a controls/ Comparab of cohorts the basis c the design analysis
Aberg et al.	2022	Sweden	Cohort	*	*	*	*	**
Almasi et al.	2009	Hungary	Case-control	*	*	*	*	*
Almroth et al.	2022	Sweden	Cohort	*	*	*	* for suicide	**
Armstrong et al.	2023	Australia	Cohort		*		*	**
Baumert et al.	2014	Germany	Cohort	*	*		*	**
Blomqvist et al.	2022	Sweden	Cohort	*	*		*	**
Conway et al.	2022	Denmark	Cohort	*	*		*	**
Dempsey et al.	2021	US	Case-control		*	*	*	

Feskanisch et al.	2002	US	Cohort		*		*	**
Guseva Canu et al.	2021	Switzerland	Cohort	*	*		*	*
Hoyer et al.	2009	Denmark	Case-control	*	*	*	*	*
Jonsson et al.	2021	Sweden	Cohort	*	*	*		**
Kolves et al.	2006	Estonia, Germany	Case-control	*	*	*	*	*
Law et al.	2014	China	Case-control	*	*			**
Lee et al.	2020	Korea	Cohort		*		*	**
Magnusson Hanson et al.	2020	Sweden	Cohort	*	*		*	**
Magnusson Hanson et al.	2023	Sweden, Finland, Denmark	Cohort	*	*		*	**
Milner et al.	2017	Australia	Case-control	*	*	*	*	*
Milner et al.	2014	Australia	Case-control	*	*	*		*
Niedhammer et al.	2021	France	Cohort	*	*		*	*
Niedhammer et al.	2022	France	Cohort	*	*	*	*	*
Ostry et al.	2007	Canada	Case-control		*	*	* for suicide	**

Padmanathan et al.	2023	UK	Cohort		*			*
Pan et al.	2023	Sweden	Cohort	*	*		*	**
Schneider et al.	2011	Germany	Case-control	*	*	*	*	**
Tsutsumi et al.	2007	Japan	Cohort		*		*	**
Wild et al.	2021	Switzerland	Cohort	*	*		*	*
